# Medical students’ perceptions of the effectiveness of module study guides: a mixed-methods study in Saudi Arabia

**DOI:** 10.3389/fmed.2026.1815080

**Published:** 2026-05-28

**Authors:** Muhammad Imran, Mukhtiar Baig, Waleed Ahmed Alghamdi, Razaz Aldemyati, Zohair Jamil Gazzaz

**Affiliations:** 1 Department of Surgery and Medical Education, Faculty of Medicine, King Abdulaziz University, Rabigh, Saudi Arabia; 2 Department of Clinical Biochemistry and Medical Education, Faculty of Medicine, King Abdulaziz University, Rabigh, Saudi Arabia; 3 Division of Psychiatry, Department of Medicine, King Abdulaziz University, Jeddah, Saudi Arabia; 4 Department of Internal Medicine, Faculty of Medicine Rabigh, King Abdulaziz University, Jeddah, Saudi Arabia

**Keywords:** assessment, gender differences, learning objectives, medical students, mixed methods research, study guides

## Abstract

**Introduction:**

This mixed-methods study examined medical students’ perceptions and satisfaction with module study guides (SGs) at the Faculty of Medicine, King Abdulaziz University, Rabigh, Saudi Arabia.

**Methods:**

Using a convergent parallel design, quantitative data were collected through structured surveys and qualitative insights through semi-structured focus groups, with both strands analyzed separately and then merged.

**Results:**

Of 490 eligible students, 255 (52%) participated, including 146 males (57.3%) and 109 females (42.7%); 95 (37.3%) were from preclinical years and 160 (62.7%) from clinical years. Overall, 111 students (43.5%) expressed satisfaction with the SGs. Students agreed that guides were available before modules (60.4%), contained clear objectives (48.2%), offered useful resources (46%) and assessment information (46%), and were easy to use (48%). Preclinical students were 2.5 times more likely to be satisfied as compared to clinical students (OR = 2.47, *p* = 0.003). Female students were more likely than male students to report satisfaction (OR = 2.07, *p* = 0.015). Qualitative findings underscored the importance of clear objectives, structured timelines, availability of digital versions of SGs, well-organized content, and alignment with assessments, while concerns were raised regarding excessive length and limited specificity. Overall, these findings indicate that students’ satisfaction with SGs is moderate and varies according to the stage of training and gender.

**Conclusion:**

The study therefore recommends that, to better support student learning and engagement, SGs should be concise, more digitally accessible, and clearly aligned with session objectives and assessment needs.

## 1 Introduction

Medical education, with multiple subjects and a lengthy curriculum, presents a unique set of demands. There are frequent assessments with diverse assessment tools. During medical education, students face many academic and non-academic challenges that affect their mental and physical health (1, 2). The shift from a teacher-centered to a student-centered learning approach demands a significant adjustment, and students need structured support, materials, and guidance to develop effective learning strategies (3). Research has shown that structured learning tools, such as guided study frameworks, play a crucial role in supporting day-to-day learning, boosting confidence, and ultimately improving academic outcomes (4). A study guide (SG) is a valuable tool that assists students in their learning journey, typically in the form printed notes. It outlines the content, instructional strategies, and self-assessment criteria (5). Well-designed SGs can promote self-directed learning (SDL), empowering students to manage their education and helping instructors in teaching and monitoring progress (6). SDL is a key component of medical education, particularly in student-centered and outcome-based curricula. The concept is grounded in established theoretical frameworks, including Knowles’ theory of andragogy, which emphasizes learner autonomy, readiness to learn, and goal-oriented learning, as well as Zimmerman’s model of self-regulated learning, which highlights processes such as goal setting, strategic planning, and self-monitoring (7, 8). Within this context, study guides may serve as structured tools that support SDL by clarifying expectations, aligning learning with assessment, and facilitating organized engagement with content. A study reported that SGs provide information about the key elements of the curriculum, such as learning outcomes (LOs), teaching strategies, and assessment methods, and also highlight the importance of a module (9).

Faculty-provided SGs can enhance self-directed learning (SDL) and improve academic performance among medical students (10). In integrated curricula, students report that SGs support day-to-day learning and foster more active engagement with material. They attribute to SGs a role in promoting regular study habits, clarifying concepts, and facilitating exam preparation (11). SGs do more than simply list syllabi and objectives; they provide structured learning pathways and deeper insight. Harden and colleagues outlined the SG format in the AMEE guide, identifying three key functions. These were to organize student learning by clarifying course structure and expectations to guide learning objectives through interactive elements used for assessment and to enrich lectures and readings with additional subject content. SGs are available in both print and electronic formats (6). Typically, SGs blend these purposes to meet the needs of learners.

Despite the reported benefits of study guides (SGs) in supporting learning, the evidence regarding their impact on objective academic outcomes remains mixed. While some studies suggest that structured learning tools can enhance student engagement and perceived understanding, these improvements do not consistently translate into measurable gains in academic performance (12, 13). Systematic reviews in medical education have similarly demonstrated that educational interventions, including structured and resource-based tools, often yield modest or variable effects on learning outcomes, highlighting the influence of contextual and design-related factors (14). The effectiveness of SGs appears to depend on their alignment with learning objectives and assessment strategies, as well as the extent to which they promote active rather than passive learning (15). Furthermore, from a cognitive perspective, overly structured learning materials may inadvertently encourage surface learning approaches or limit deeper cognitive engagement if not carefully designed (16). These findings suggest that SGs are not inherently effective but rather function as context-dependent educational tools whose impact is mediated by instructional design, implementation, and learner engagement.

SGs play a crucial role in global medical education, yet research on their effectiveness and student satisfaction in Saudi Arabia remains limited, particularly within integrated curricula. A study at King Abdulaziz University conducted over a decade ago focused on developing and evaluating an SG template for an integrated cardiovascular module (17). However, there is a notable gap in the literature regarding the broader use, effectiveness, and student perspectives on SGs in Saudi medical schools.

This study aimed to address this gap by exploring the experiences of students at the Faculty of Medicine in Rabigh (FoMR). FoMR follows a hybrid, integrated, modular curriculum, in which each module is supported by a standard SG outlining key module information. The hybrid nature of the curriculum refers to the combination of traditional face-to-face teaching with student-centered learning approaches, including problem-based learning, small-group discussions, and self-directed learning activities. Integration is achieved through a system-based design in which basic and clinical sciences are taught in a coordinated manner within each module, rather than as isolated disciplines. The curriculum is delivered in modular blocks, each focused on a specific organ system or theme, with clearly defined learning outcomes, aligned teaching strategies, and structured assessment methods. The SG directs students to relevant resources and assists them in achieving the learning objectives. As part of ongoing curriculum reform, this study examines students’ perceptions of and satisfaction with SGs at FoMR, King Abdulaziz University (KAU), Rabigh. Insights from this work are expected to facilitate improvements in future SGs and contribute to broader curriculum development.

## 2 Methods

### 2.1 Study design and setting

This mixed-methods study employed a convergent parallel design, in which both quantitative and qualitative data were collected concurrently and analyzed independently before being merged for interpretation. The study was conducted at FoMR, KAU, Rabigh. The research proposal was approved by the Research Ethics Committee, Unit of Biomedical Ethics, Faculty of Medicine, Rabigh, under reference number 24030. A mixed-methods approach allows for a more comprehensive understanding by combining the breadth of quantitative data with the depth of qualitative insights (18). Quantitative data were obtained through structured surveys, measuring students’ perceptions and satisfaction with SGs. Qualitative data were generated from semi-structured focus groups, providing deeper insights and suggestions. The study was conducted over a five-month period.

In Saudi Arabia, medical education lasts 6 years, with the first year being preparatory and medical courses starting in the second year. Data collection included students from the second to sixth year. FoMR is a 15-year-old medical college located in the small industrial town of Rabigh, approximately 160 kilometers from Jaddah (19). Each course and module at FoMR has a faculty-prepared SG distributed before the start of the course, and these SGs have been in use since the college was established (20). Clinical rotations begin in the fourth year; therefore, students in the fourth year and above are classified as the clinical group.

Participants included male and female undergraduate medical students from the second to sixth years. This was a census survey in which all medical students at FoMR were invited to participate. As the entire student population was targeted, a formal sample size calculation was not required. To explore potential non-response bias, respondents were compared with the overall student cohort using available institutional data on gender and academic year. Academic performance data were not collected for this study and therefore were not included in the analysis.

### 2.2 Ethical considerations

All procedures involving human participants were conducted in accordance with the ethical principles stated in the Declaration of Helsinki. All enrolled students were eligible to participate regardless of academic performance. Written informed consent was obtained from all participants prior to inclusion in the quantitative component of the study, and students who did not provide consent were excluded. For the qualitative component, written informed consent was obtained from all participants before participation, and verbal consent was reconfirmed prior to audio recording. Participants were reminded that their participation was voluntary and they that they could withdraw from the study at any stage without any academic or personal consequences.

### 2.3 Recruitment, tools, and data collection

A self-administered questionnaire was developed for the quantitative component, drawing on previously published and peer-reviewed instruments (9, 21). Permission was obtained from the original authors to use and adapt relevant items. In addition, the authors (MI, MB) developed a small number of context-specific items to tailor the tool to the local educational environment. The questionnaire consisted of both closed-ended items, including 5-point Likert scale questions, and a few open-ended questions to allow for further elaboration. The 5-point Likert scale responses were collapsed into three categories (“agree,” “neutral,” and “disagree”), with “strongly agree” and “agree” combined and “strongly disagree” and “disagree” combined. This approach was adopted to simplify interpretation, enhance clarity of presentation, and facilitate comparison across items by focusing on the overall direction of responses. Such categorization is commonly used in survey-based studies when the primary objective is to identify general trends rather than fine distinctions in response intensity. Three experts in medical education reviewed the questionnaire for face and content validity. Our pilot study was conducted with twenty students (not included in the final analysis), and necessary revisions were made based on their feedback. The reliability of the final instrument was assessed using Cronbach’s alpha, yielding a coefficient of 0.93, which indicates excellent internal consistency. The final questionnaire was converted into a Google Form and distributed online via students’ WhatsApp groups and their official e-mail accounts. Participation was voluntary and anonymous, and informed consent was obtained electronically prior to participation.

To complement this survey data and explore students’ experiences and perceptions in greater dept., four focus group discussions (FGDs) were conducted. These were organized to ensure representation across academic years and gender. The groups included:

Two FGD sessions with preclinical students (second and third years): one male group and one female group.

Two FGD sessions with clinical students (4th, 5th, and 6th years): one male group and one female group.

Each FGD session consisted of five to seven participants and lasted approximately 40 to 50 min. A semi-structured interview guide, developed by the research team, was used and piloted prior to implementation. The discussion focused on students’ experiences with current SGs, perceived usefulness, suggestions for improvement, and views on structure, content, and format. All FGDs were audio-recorded and later transcribed verbatim. Transcripts were first returned to the respective student groups for validation to ensure accuracy and authenticity and were then reviewed by the research team for consistency and completeness. Data were analyzed using a rigorous thematic analysis approach involving iterative reading of the transcripts, systematic coding of meaningful segments, organization of codes into subthemes, and synthesis of these subthemes into overarching themes to capture the essence of participants perspectives, as described by Braun and Clarke (22). Recording was conducted independently by two researchers (MI, MB) to enhance inter-coder reliability, with discrepancies resolved through consensus. Themes were supported by verbatim quotations to illustrate key points and preserve participants’ voices. An inductive, data-driven approach was adopted, allowing themes to emerge directly from the participants’ narratives without restriction by a predefined theoretical framework.

**Figure 1:**
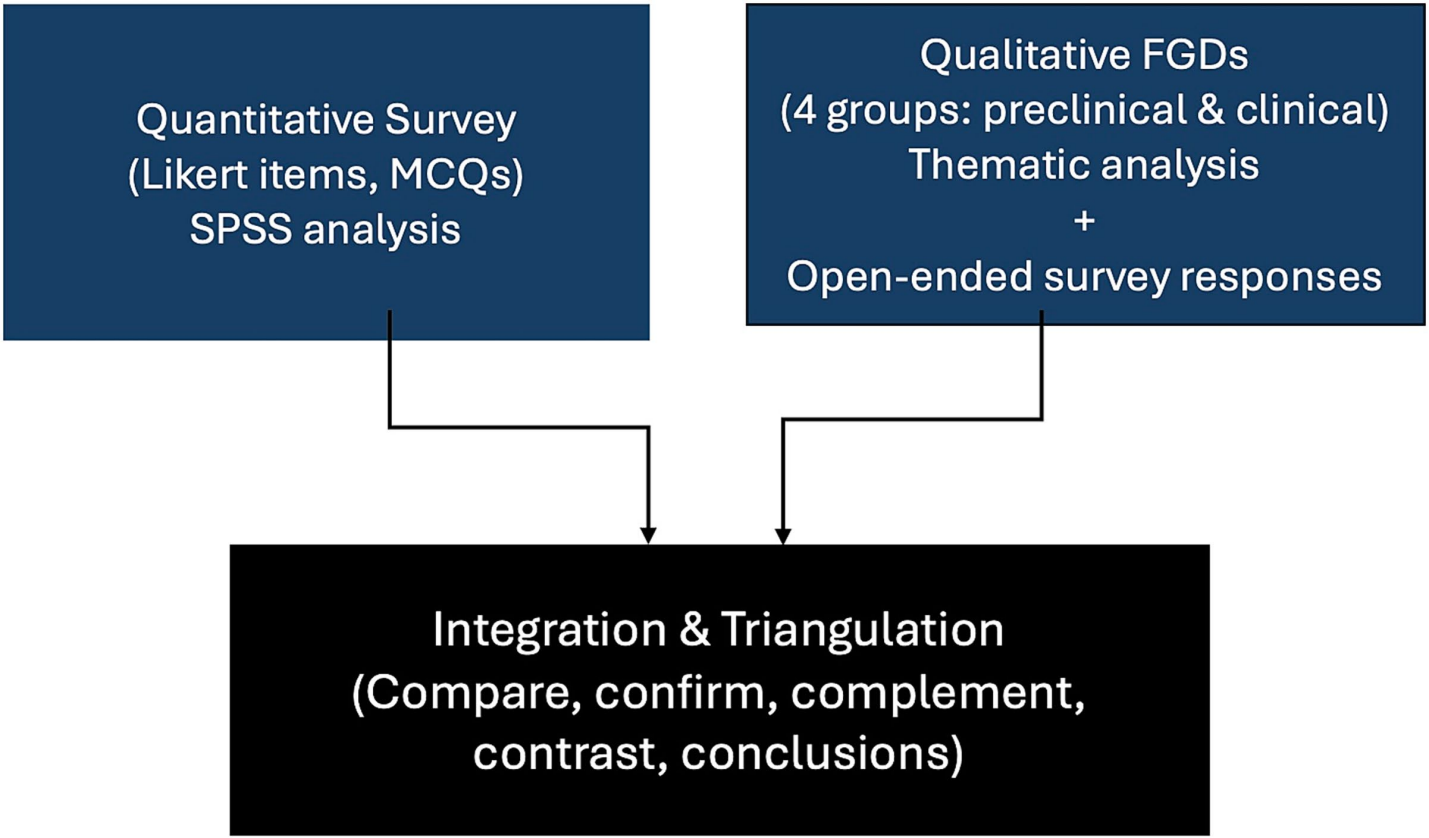
Triangulation of quantitative and qualitative findings.

Both quantitative and qualitative data were collected concurrently as part of a convergent parallel mixed-methods design, with equal priority given to both components. The quantitative strand provided a broad assessment of students’ perceptions, while the qualitative strand offered in-depth insights into their experiences. Thematic saturation was achieved by the fourth focus group, as no new codes or themes emerged and responses became repetitive, indicating adequate depth and coverage of the data. Findings from both components were compared and contrasted during the interpretation phase. Triangulation was employed to validate results, enhance credibility, and ensure a comprehensive understanding of students’ perceptions of the use and improvement of study guides, allowing identification of areas of convergence, complementarity, and divergence across datasets. Specifically, key quantitative findings were aligned with corresponding qualitative themes, and areas of agreement, complementarity, and divergence were identified through iterative comparison. This process enabled the qualitative data to contextualize and explain survey results, particularly in areas where quantitative findings required deeper interpretation. Figure 1 illustrates the triangulation process integrating quantitative and qualitative data. All data were anonymized, securely stored, and accessible only to the research team.

### 2.4 Statistical tests

Continuous variables describing participants’ general characteristics are reported as means and standard deviations, whereas categorical variables are presented as frequencies and percentages. Students’ responses to the SG-related items are similarly summarized using frequency and percentage distributions. The relationships between GPA, academic classification (preclinical and clinical), and gender with students’ satisfaction with the SG were analyzed using the chi-square test.

By converting student satisfaction into “satisfied,” “neutral,” and “not satisfied” with the SGs, their association was analyzed using ordinal regression. A *p*-value less than 0.05 was considered significant. The ordinal regression was employed because of the ordered nature of the satisfaction variable. The proportional odds assumption was tested using the Test of Parallel Lines. As the assumption was met (*p* > 0.05), ordinal logistic regression was selected for the final analysis.

## 3 Results

### 3.1 Quantitative

A total of 255 out of 490 (52%) medical students participated in this survey, comprising 146 males (57.3%) and 109 females (42.7%). The average age was 22.23 ± 1.90 years. There were 95 students (37.3%) from the preclinical years (second and third years) and 160 students (62.7%) from the clinical years (fourth, fifth, and sixth years). The mean GPA was 4.17 ± 0.58, and 211 students (83%) were high achievers (GPA > 3.5). Overall, 111 students (43.5%) reported being satisfied with the SG, and most students preferred using digital versions (212 students, 83%) (Table 1).

**Table 1:** General characteristics of the study participants (*n* = 255).

Variables	*N* (%)
Gender	
Males	146 (57.3)
Females	109 (42.7)
Age in years (mean ± SD)	Mean 22.23 ± 1.90
Study year	
Second year	38 (14.9)
Third year	57 (22.4)
Fourth year	57 (22.4)
Fifth year	48 (18.8)
Sixth year	55 (21.8)
Academic classification	
Preclinical (second and third year)	95 (37.3)
Clinical (fourth, fifth, sixth year)	160 (62.7)
GPA (mean ± SD)	4.17 ± 0.58
GPA grouping	
Group A GPA 2–3.5 (low achievers)	44 (17.3)
Group B GPA > 3.5 (high achievers)	211 (82.7)
Students’ perceptions of their academic performance	
Excellent	55 (21.6)
Good	113 (44.3)
Average	72 (28.2)
Below average	15 (5.9)
Students’ perception about SG	
Satisfied	111 (43.5)
Neutral	104 (40.8)
Not satisfied	40 (15.7)
Preference of SG version	
Digital (e.g., PDF, online)	212 (83.1)
No preference	29 (11.4)
Printed (hardcopy)	14 (5.5)

*N* (%) = frequency (percentage), GPA, grade point average, SD, standard deviation, SG, study guide.

Students gave diverse responses about SG, including availability before the start of the module (60.4% agreed), clear learning objectives (48.2%), helpful resources (46%), useful assessment information (46%), and ease of use (48%). Areas requiring improvement include the adequacy of external resources (44%), infrequent usage (reported by only 43%), user-friendliness (51%), and the clarity of examination rules (43.5%) (Table 2).

**Table 2:** Students’ responses to different study guide related questions.

Variables	Disagree *N* (%)	Neutral *N* (%)	Agree *N* (%)
1. The study guide was given for prior reference before the beginning of the module.	40 (15.7)	61 (23.9)	154 (60.4)
2. The study guide was easy to follow.	49 (19.2)	84 (32.9)	122 (47.8)
3. The study guide was user friendly	49 (19.2)	77 (30.2)	129 (50.6)
4. The introduction helped us realize the importance of the module.	47 (18.4)	95 (37.3)	113 (44.3)
5. Learning objectives were clearly stated.	35 (13.7)	97 (38)	123 (48.2)
6. Learning strategies identified in the guide matched college schedule.	46 (18)	99 (38.8)	110 (43.1)
7. List of resources was helpful for self-learning.	35 (13.7)	103 (40.4)	117 (45.9)
8. Links to external learning resources were adequate for my learning needs.	27 (10.6)	116 (45.5)	112 (43.9)
9. Identified resource person made it easy to seek guidance regarding my concerns.	31 (12.2)	109 (42.7)	115 (45.1)
10. Information on the assessment tools (MCQ, OSPE, OSCE, others) was useful in preparing for exams.	52 (20.4)	85 (33.3)	118 (46.3)
11. The study guide was helpful in the learning process throughout the module.	46 (18)	98 (38.4)	111 (43.5)
12. Examination rules were clearly stated.	43 (16.9)	101 (39.6)	111 (43.5)
13. Study guide did not support learning for this module.	92 (38.8)	85 (33.3)	71 (27.8)
14. I frequently use the study guide during the module.	66 (25.9)	78 (30.6)	111 (43.5)
15. Study guide should include space for student’s reflection	53 (20.8)	*n* (33.7)	116 (45.5)

Questions were answered on a 5-point Likert scale. For simplicity in data representation, the categories “strongly agree” and “agree” were combined into “agree”, as were “disagree” and “strongly disagree” into “disagree”. N (%) = frequency (percentage).

An ordinal logistic regression analysis showed that preclinical students were 2.47 times more likely to be satisfied compared to clinical students (OR = 2.47, *p* = 0.003). Female students were 2 times more likely to be satisfied compared to males (OR = 2.07, *p* = 0.015). GPA was not significantly associated with satisfaction (Table 3).

**Table 3:** Ordinal logistic regression analysis of factors associated with student satisfaction with study guides.

Variable	Coefficient	S.E.	Wald *χ*^2^	OR	*p*-value	95% CI (lower–upper)
GPA						
>3.5	1					
2–3.5	− 0.016	0.32	0.002	0.98	0.961	−0.636 to 0.605
Gender						
Male	1					
Female	0.73	0.30	5.91	2.07	0.015	1.41–1.312
Academic classification						
Clinical	1					
Preclinical	0.91	0.31	8.57	2.47	0.003	0.299–1.51

Outcome variable: satisfaction (1 = Not satisfied, 2 = Neutral, 3 = Satisfied). Model estimated using ordinal logistic regression with a logit link. GPA, grade point average.

### 3.2 Qualitative

Six interconnected themes emerged from the analysis of four FGDs (Table 4). Students perceived current SGs as having clear objectives, structured timelines, and well-organized content, however, they also identified limitations, including excessive length and, in some instances, a lack of specific learning objectives. One participant explained, “A shorter and focused SG keeps me on track, but when it’s dozens of pages long, I find it overwhelming and set it aside” (FGD3P4). This view was shared by several other participants.

**Table 4:** Students’ perceptions and suggestions about study guides: themes, sub-themes, and verbatim quotes.

Themes	Sub-themes (with definitions)	Representative verbatim quotes
Perceptions of current study guides (positive and negative)	Clarity of objectives and expectations (clearly stated learning outcomes and scope). Structured timelines and content (organized schedules and lecture outlines). Limitations and gaps (missing objectives, excessive length, or unclear focus).	“Mostly, the objectives are clear, so I know exactly what I’m expected to learn.” “The timeline makes it easy to follow what’s next.” “Some study guides are more than 50 pages long, which really discourages me.” “A few modules had only topics without any objectives.”
Practical utility for learning and planning	Weekly scheduling (timetable used as a planning tool). Monitoring assignments and deadlines (awareness of key submission dates). References for self-study (use of provided references for independent learning).	“One important point: I use the timetable in the study guide to plan my study week.” “It tells me the deadlines and mark distribution, which helps me prepare.” “The references in the guide save my time searching for resources.”
Structural and content relevance	Alignment with lectures and cases (study guide content reflects what is taught). Adequacy of detail (balance between depth and conciseness). Integration with assessment (explicit links to exam format and assessment blueprints).	“Sometimes the guide just lists topics, not the learning objectives.” “It would be great to have a blueprint showing how each topic is assessed.” “Some guides matched the lectures exactly, which made revision easy.”
Format, accessibility, and delivery (digital preference)	Electronic preference (greater usability and portability in digital format). Embedded resource links (direct access to readings, videos, or online tools). Ease of navigation (clear structure for quick reference).	“Electronic is easier to navigate and can have clickable links.” “Some guides had links to materials and believe me that was very helpful.” “Printed guides are harder to update; online versions can be revised quickly.”
Desired enhancements for future study guides	Clinical schedules and skill lists (information on practical opportunities and competencies). Visual aids and procedural videos (diagrams, flowcharts, and demonstrations). Interactive student activities (self-assessment tools and quizzes).	“If we had the doctors’ clinic times, we could track them easily.” “Adding flowcharts or videos for procedures would help a lot.” “It would be useful to have questions at the end of each section for revision.”
Patterns of engagement with study guides	Selective reading (students focusing only on high-priority sections). Limited engagement with optional activities (low usage of self-study prompts). Recovery after missed lectures (guide as a tool for catching up).	“I only check the objectives and deadlines; the rest I skip.” “If I miss a lecture, the study guide tells me what I need to cover.” “Sometimes I ignore the activities in the guide; I just focus on core material.”

Students emphasized the practical role of SGs in managing their learning, using them to plan study schedules, track deadlines, and access recommended readings. One participant reflected, “The schedule in the guide is like my semester plan; it helps me see what’s coming up and when I need to submit work” (FGD1P4). The structural organization and content relevance of SGs were considered critical, with participants underscoring the importance of alignment with teaching sessions, adequate detail, and explicit links to assessment methods. One student commented, “If I know how this section will be examined, I can plan my studies better; without that description I feel I am studying blindly” (FGD2P1). Similar sentiment was expressed across multiple FGDs. Some participants expressed concern about the lack of clear assessment blueprints.

In terms of format, accessibility, and delivery, students showed a strong preference for electronic SGs, particularly valuing the ease of access on mobile devices. As one participant noted, “I can open the SG on my phone anytime, anywhere, and follow the links instantly; carrying a printed version feels unnecessary and it quickly goes out of date” (FGD4P7). Another participant endorsed, “Honestly, electronic is better we are in the 21st century and in the AI era so paper versions do not really make sense anymore” (FGD4P3). The view was supported by many students in different FGDs. When discussing potential enhancements, students suggested incorporating clinical timetables, lists of required skills, visual learning aids, and interactive elements. One participant proposed, “If the guides included clinic days for different specialties and maybe a few demonstration videos, it would be far more engaging and practical” (FGD1P3), an idea that resonated strongly among peers. Finally, patterns of engagement differed, with some students using SGs consistently, while others referred mainly to key sections such as objectives, deadlines, and grading criteria, particularly when catching up after missed sessions.

Analysis of open-ended survey responses revealed several key points that supplemented the quantitative findings. Students emphasized the value of clear learning objectives, well-organized structure, and easy accessibility of SGs, reflecting the most frequently selected survey elements. They also highlighted challenges, including excessive length, unclear objectives in some sessions, and limited guidance regarding assessment. Many students proposed practical improvements, such as concise summaries, digital versions, explicit links to readings and videos, inclusion of clinical timetables and skill lists, and interactive features like practice questions. These responses closely aligned with the themes identified in the FGDs, providing additional depth and context to students’ perceptions and suggestions for enhancing SGs.

### 3.3 Triangulation of study guide perceptions

**Figure 2:**
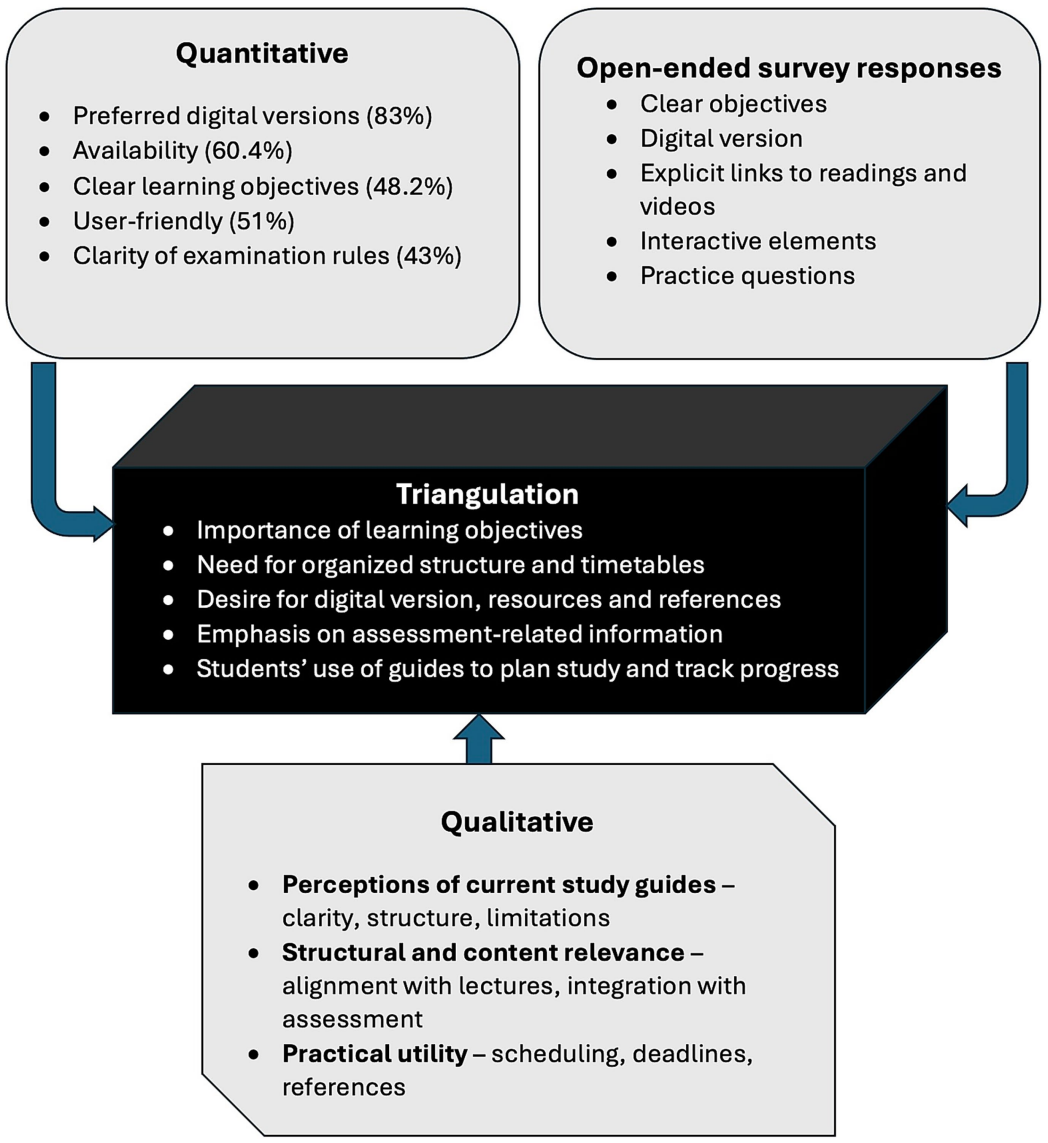
Integration of quantitative, qualitative, and open-ended survey responses.

Figure 2 shows how findings from quantitative surveys, open-ended responses, and focus group discussions aligned. The triangulated results revealed strong agreement on the key features of effective SGs, including clearly defined objectives, a well-organized structure, and guidance related to assessments. In addition, the qualitative data added depth by highlighting unique suggestions such as incorporating interactive elements, providing concise summaries, and improving accessibility through digital versions.

## 4 Discussion

The present study observed that although a considerable proportion of students reported satisfaction with SGs, this did not represent a majority, and their feedback highlighted several important areas for improvement. When asked about specific features, many responses fell within the moderate-to-low range. For instance, only about 60% found the SG available early enough, and fewer than half stated it had clear learning objectives (48%), helpful resources (46%), useful assessment details (46%), or adequate external references (44%). This suggests that although overall satisfaction is relatively positive, significant improvement could be made across various sections of the SG to better meet students’ needs. These findings likely reflect the diverse needs and expectations among students. One key insight is that SG is not being used frequently by a significant number of students (43%), even though it is readily available. This lower engagement could stem from a few factors: some students may find it redundant, preferring to rely on other materials, such as online sources, handouts, or textbooks. Others may not see it as helpful for exam preparation, or feel it is not well aligned with how they learn or what they are assessed on. This aligns with observations from Mathai et al. (23), who noted that medical students often find formal resources lacking in depth and organization, while turning to more concise, informal resources for better understanding and retention (23). This finding suggests that availability alone does not ensure meaningful utilization and points toward potential gaps at the level of design, communication, and curriculum integration. The qualitative findings support this, with students emphasizing the importance of alignment and specificity in enhancing usefulness.

The present study showed that, within the resource domain, 46% of students perceived the resources provided in the SGs as helpful, while 44% considered the available external resources adequate. Overall, these findings suggest that many students viewed the learning resources as insufficient. Consistent with these results, a recent Saudi study examining the strengths and challenges of clinical pharmacology curriculum delivery reported limited access to up-to-date and comprehensive learning resources, along with inadequate opportunities for hands-on and practical application (24). Another Saudi study found that system quality, information quality, and interaction quality, factors closely related to clarity, resource availability, and ease of use, had a strong influence on students’ satisfaction. Although this study did not focus specifically on SGs, its findings highlight key elements that shape satisfaction with self-directed and online learning (25). Taken together, these observations suggest that well-designed SGs with accessible and high-quality resources are likely to enhance students’ satisfaction.

The current study found that preclinical students reported higher satisfaction with SGs compared to clinical students. Several factors may explain this difference. While this may reflect differences in learning requirements across phases, a more nuanced explanation emerges when considered alongside the qualitative findings. Preclinical students primarily study basic science subjects, which are often better supported by structured resources such as SGs. As beginners in the medical program, they may also find SGs particularly helpful in guiding them through the curriculum and clarifying expectations for each subject or module. In contrast, clinical students spend much of their time in hospital settings, where learning is largely patient-centered and context-driven. Their education relies heavily on real-time clinical encounters, bedside teaching, and direct interaction with patients, which may reduce their reliance on structured resources such as SGs. Additionally, clinical rotations involve multiple competing demands, including patient care responsibilities, ward duties, and shift work, which may further limit the perceived usefulness of SGs. It is also likely that clinical students feel that existing SGs do not adequately reflect the practical and experiential nature of their clinical learning. Consistent with the present findings, a previous study from King Abdulaziz University reported differences between preclinical and clinical students in their approaches to professional studies and use of spare time (19). Similarly, findings from a simulation-based learning study showed that preclinical students expressed higher satisfaction with structured learning supports compared with their clinical counterparts (26). The qualitative themes of alignment and specificity further support this interpretation. Students highlighted the usefulness of study guides when objectives were clearly aligned with teaching and assessment, and when content was specific and focused. However, clinical students expressed a need for more contextualization, prioritization, and guidance on application, suggesting that the current format of study guides may not adequately address the demands of clinical learning. This indicates that while study guides are effective in early phases, their design may require adaptation to better support higher levels of cognitive processing and clinical reasoning.

The strong student preference for a digital version of the study guides in this study likely stems from features like searchability, mobile access, and verified links to readings and videos. This aligns with a broader shift toward digital study habits among medical undergraduates, who often prioritize immediate and portable resources. Digital delivery thus enhances accessibility while also allowing for the integration of interactive components and timely updates (27, 28). Support for this argument comes from a recent nationwide survey of Swedish medical students, which found that digital resources, particularly curated videos and curriculum-specific flash cards, were widely used and appreciated for their availability, flexibility, and effectiveness. Such well-designed electronic guides appear to function as structured learning pathways rather than mere objective listings (29).

Female students were more satisfied than male students, a difference that may reflect different learning styles. It is possible that the organization and structure of the SGs align more closely with the learning preferences of female students. Socio-cultural or institutional factors could also influence how male and female students approach their education and engage with learning materials. However, since this difference was only borderline significant, the results are not conclusive. These observations are consistent with a Saudi study, which found that female students tend to exhibit stronger study habits, including a preference for clear guidelines, a task-oriented approach, and greater time devoted to studying compared to males (20).

In the present study, students clearly expressed a preference for learning objectives defined at the level of individual sessions, along with transparent assessment information, including mark distribution and examination focus. They emphasized the need for clearly organized content and easy access to relevant learning resources. A digital version of SGs, with embedded links, was strongly favored. Students described using SGs both as planning tool throughout the module and as a quick reference when preparing for assessments. They also proposed several practical enhancement suggestions, such as interactive questions, clinical timetables, and visual aids, to increase the overall usefulness of SGs. These findings are consistent with recent studies showing that students depend heavily on clearly signposted objectives and curated resources when organizing their learning, and that the type of resources they select can influence study, behaviors and academic performance (30). Earlier work conducted nearly two decades ago, similarly, reported that students generally preferred timetable-based SGs, although some favored problem-based or outcome-based formats (31). Another study concluded that self-study guides were perceived as valuable tools for achieving learning goals, particularly when used alongside effective planning, time management, and timely support-seeking behaviors. Personalized feedback further enhanced their role in promoting independent learning (32). In line with these observations, the quantitative findings of the present study demonstrated that learning objectives and recommended readings were among the most highly valued elements of SGs. This was reinforced by in-depth discussions during the FGDs and aligns with recent evidence linking medical students’ resource choices to learning outcomes (33).

Our findings highlight the importance of aligning learning objectives, teaching activities, and assessments, a principle strongly supported by contemporary curriculum research. Explicit alignment is seen as essential for fostering coherent study strategies and deeper learning. Students reported clearer expectations and greater engagement when instructors made objectives visible and connected them to formative tasks, as evidenced in recent course-level implementations (27, 34). The conciseness and navigability of the SGs also proved to be a practical necessity. Excessively long guides were frequently described as overwhelming, often leading students to read only the sections perceived as most essential. This observation is consistent with cognitive load theory and related empirical work, which indicate that poorly structured or wordy materials increase extraneous cognitive load, thereby hindering a student’s ability to process complex clinical information. In contrast, brief and well-organized SGs reduce this extraneous load and support learning more effectively (35). A dominant theme in the feedback was the desire for greater assessment transparency. Students expressed a clear preference for a test blueprint, or, at a minimum, for explicit details on the number of questions and the weighting assigned to each lecture or topic. This preference aligns with established best practices in assessment design, where blueprinting is recommended to ensure content validity and focus student preparation. Adopting practical, resource-sensitive approaches to blueprinting could enable course teams to provide students with useful assessment outlines (36, 37).

The findings for this study indicate that several structural and content-related enhancements are needed in the current SGs. Taken together, the triangulated evidence points to a set of practical, low-cost improvements that could be implemented without major curriculum disruption. These include adopting a digital SG format, alongside improvements in instructional design, such as clearly stating learning objectives at the level of individual sessions, providing transparent assessment blueprints, and incorporating curated links to key resources alongside brief practical activities. The use of visual learning aids, such as flow charts and mind maps, may further support comprehension by reducing extraneous cognitive load. These recommendations are consistent with best practices in instructional design and assessment and are feasible to implement using existing learning management systems (35–37). However, the extent and manner of structuring require careful consideration.

While these enhancements may improve the usability and effectiveness of SGs, it is important to consider potential unintended consequences. Overly detailed or highly structured SGs may inadvertently promote learner dependency, potentially limiting the development of self-directed learning (SDL) skills. In medical education, SDL requires learners to identify their own learning needs, engage with diverse resources, and regulate their learning strategies (7). Excessive reliance on predefined content and guidance may reduce opportunities for exploration and critical thinking, thereby encouraging more surface-oriented learning approaches. From a cognitive perspective, although structured materials can reduce extraneous cognitive load (38), they must be carefully designed to avoid constraining deeper processing and learner autonomy. Similarly, highly guided instructional approaches, if not balanced appropriately, may limit active knowledge construction (16, 39). Therefore, SGs should be conceptualized not as prescriptive tools, but as flexible frameworks that scaffold learning while still encouraging independent inquiry and active engagement.

The study presents notable methodological strengths. Its primary contribution is the novel application of a mixed-methods triangulation approach to assess students’ perceptions and satisfaction regarding the SGs, combining quantitative surveys, qualitative written responses, and FGDs. This design ensured comprehensive insight, with the quantitative component revealing population-wide trends and the qualitative components providing depth, contextual richness, and individual narratives. The FGDs facilitated collective reflection, generating perspectives complementary to individual data. Such triangulation reinforces the trustworthiness and robustness of the results by allowing for cross-validation. Although the findings are especially pertinent to the FoMR, they may hold relevance for comparable medical education institutions.

This study has several limitations that should be acknowledged. Although a census-based survey approach was employed, the overall response rate was 52%, which may introduce the possibility of non-response bias and should be considered when interpreting the findings. A partial assessment of non-response bias was performed using available institutional data, comparing respondents with the overall student cohort in terms of gender and academic year; respondents were broadly comparable in these characteristics. However, academic performance data were not available for analysis, and therefore residual non-response bias cannot be fully excluded. In addition, it is possible that students with greater engagement or more positive perceptions of study guides may have been more likely to participate, which may have influenced the reported findings. In addition, as the study was conducted at a single institution within one region, the generalizability of the results to other medical schools may be limited. Furthermore, in the qualitative component, self-selection of participants may have introduced selection bias, with a potential over-representation of students who were more motivated or engaged with the topic. In addition, reliance on self-reported data may have introduced reporting bias, which could influence the accuracy of perceived experiences and behaviors. Finally, collapsing Likert-scale responses, while improving interpretability, may have reduced statistical sensitivity and limited the ability to distinguish between varying degrees of agreement or disagreement. As a result, potentially meaningful differences between moderate and strong responses may not have been fully captured. Future research should address these limitations by implementing and evaluating the proposed study guide enhancements across multiple institutions and examining their impact on student learning behaviors and assessment outcomes using more robust, longitudinal designs.

## 5 Conclusion

The findings of this study suggest that students consistently value clearly defined session-level learning objectives, transparency regarding assessments, concise and well-organized content, and digital versions of SGs. In response to these preferences, the adoption of a brief, digital, and blueprint-aligned SG template that incorporates curated resources and interactive practice questions may be well received by students. These measures are aligned with contemporary educational theory and best practices in assessment. Strengthening the design, curricular integration, and adaptability of SGs across the program may enhance their educational value.

## Data Availability

The raw data supporting the conclusions of this article will be made available by the authors, without undue reservation.
